# Supplementary material for: A disinhibitory nigra-parafascicular pathway amplifies seizure in temporal lobe epilepsy
Source: Nat Commun. 2020 Feb 17;11:923. doi: 10.1038/s41467-020-14648-8 (PMC7026152; doi:10.1038/s41467-020-14648-8)
Supplement: Supplementary file 1 — Supplementary Information [file 41467_2020_14648_MOESM1_ESM.pdf]

SUPPLEMENTARY INFORMATION

A disinhibitory nigra-parafascicular pathway amplifies seizure in temporal lobe epilepsy

Chen et al.

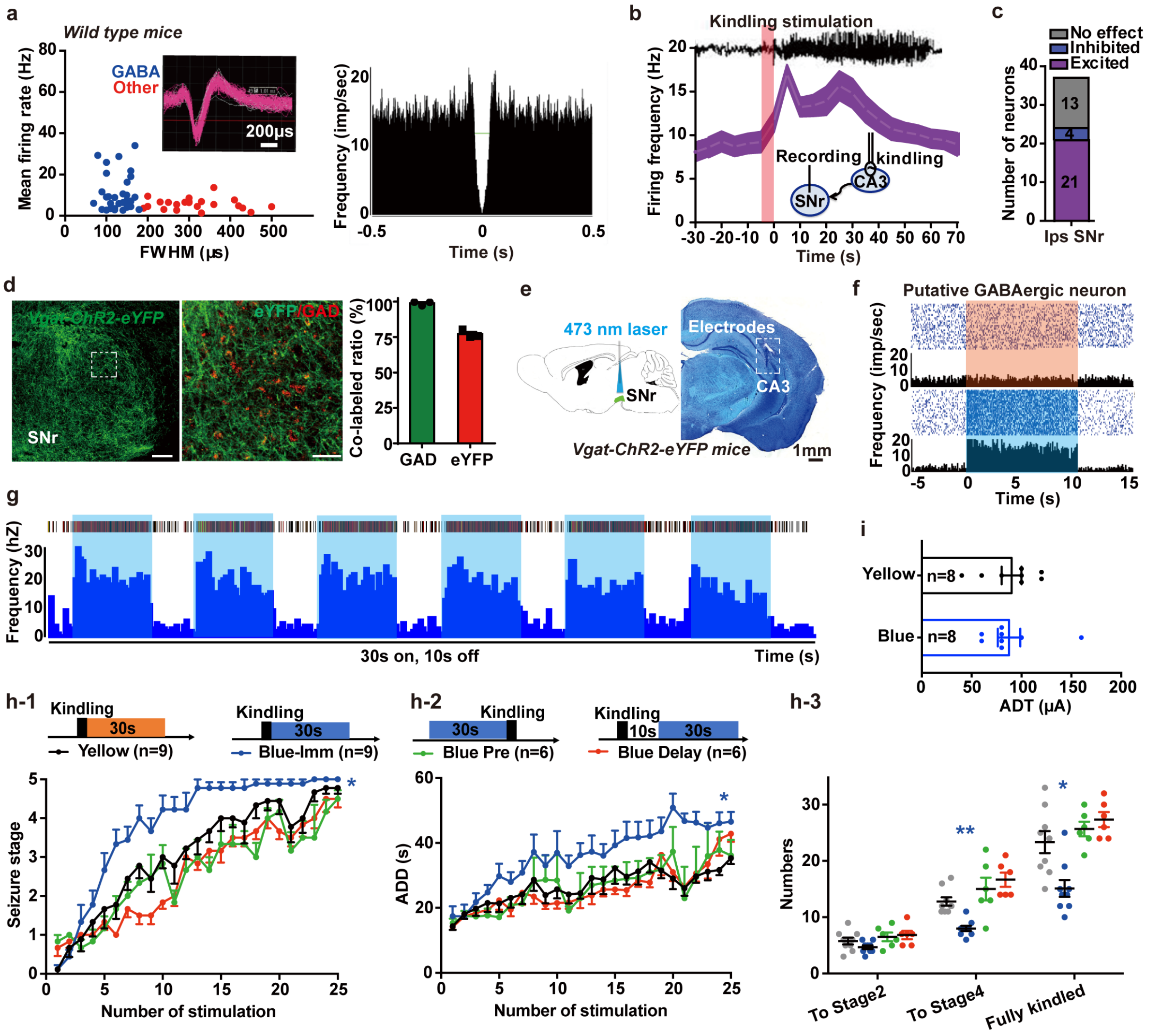

Supplementary Figure 1. Activation of SNr GABAergic neurons accelerates kindling progression in TLE. (**a**) Classification of SNr GABAergic neurons. Left, putative GABAergic neurons have narrow waveform (full width half maximum, FWHM<200 μs) and high mean firing rate (9.24 ± 1.32 Hz, 38 neurons recorded in the ipsilateral SNr from 3 wildtype mice). Insert, representative spike waveform; right, corresponding flat autocorrelation. (**b**) Local field potentials (LFPs) and average firing rates of activated clusters of SNr GABAergic neurons during hippocampal kindling-induced seizures. Insert, scheme of experiment for kindling stimulation in the CA3 and *in vivo* single-unit recordings in the ipsilateral SNr. (**c**) Statistic of responses of recorded SNr GABAergic neurons ipsilateral to kindling site during kindling-induced seizures. (**d**) Left, representative images of the SNr from a *Vgat-ChR2-eYFP* mouse, showing the overlap of ChR2-eYFP and glutamate decarboxylase (GAD). Scale bar, 50 μm. Right, quantification of the percentage of ChR2-eYFP neurons that expressed GAD from 3 *Vgat-ChR2-eYFP* mice (repeated 4 times per mouse). (**e**) Scheme of experiment for photostimulation in the SNr and placement of electrodes for kindling stimulation in the CA3 of *Vgat-ChR2-eYFP* mice. (**f**) Representative peri-event raster histograms showing the firing of the same SNr GABAergic neuron in response to blue-light stimulation (473 nm, 20 Hz, 10 ms, 5 mW, 10 s on-off cycle) and yellow-light (589 nm, continuous light, 5 mW, 10 s on-off cycle). (**g**) Representative peri-event raster histogram showing the firing rate of the SNr GABAergic neuron in response to blue-light stimulation (473 nm, 20 Hz, 10 ms, 5 mW, "30s-on, 10s-off" cycle) in an awake *Vgat-ChR2-eYFP* mouse. (**h, i**) Effects of optogenetic

activation of SNr GABAergic neurons with different photos-timulation timing on the development of seizure stage (**h-1**) and afterdischarge duration (ADD, **h-2**), number of stimulations needed to reach each stage (**h-3**) and afterdischarge threshold (ADT, **i**) in hippocampal kindling model. Yellow group means yellow light stimulation immediately after each kindling stimulation, Blue-Imm group means blue light stimulation immediately after each kindling stimulation, Blue-Pre group means blue light stimulation before each kindling stimulation, while Blue-Delay group means blue light stimulation after each kindling stimulation with a 10s delay. Align-and-rank data for a nonparametric ANOVA followed by *post hoc* Dunn's test was used for **h-1,** General linear model with repeated measures followed by *post hoc* Dunn's test was used for **h-2**, and Mann-Whitney U test with *post hoc* Dunn's was used for **h-3**, *P<0.05, **P<0.01 compared with yellow-light control. The number of mice used in each group is indicated in figure. All the data are presented as mean ± S.E.M.. Source data are provided as a Source Data file.

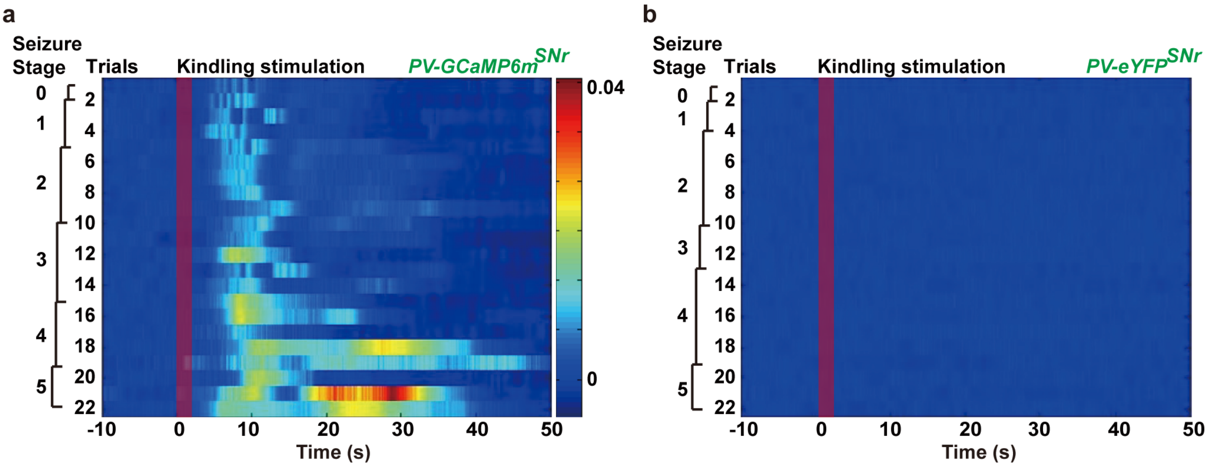

Supplementary Figure 2. Calcium signal of SNr PV neurons gradually increases during kindling acquisition. (**a**) Representative heatmap of calcium signals aligned to the initiation of kindling stimulations from a *PV-GCaMP6m*^{SNr} mouse. (**b**) Representative heatmap of calcium signals aligned to the initiation of kindling stimulations from a *PV-eYFP*^{SNr} mouse. Each row represents the calcium signal of each seizure stage during kindling acquisition. Color scale indicated ΔF/F, warmer colors indicated higher fluorescence signal. The red rectangles represent kindling stimulation.

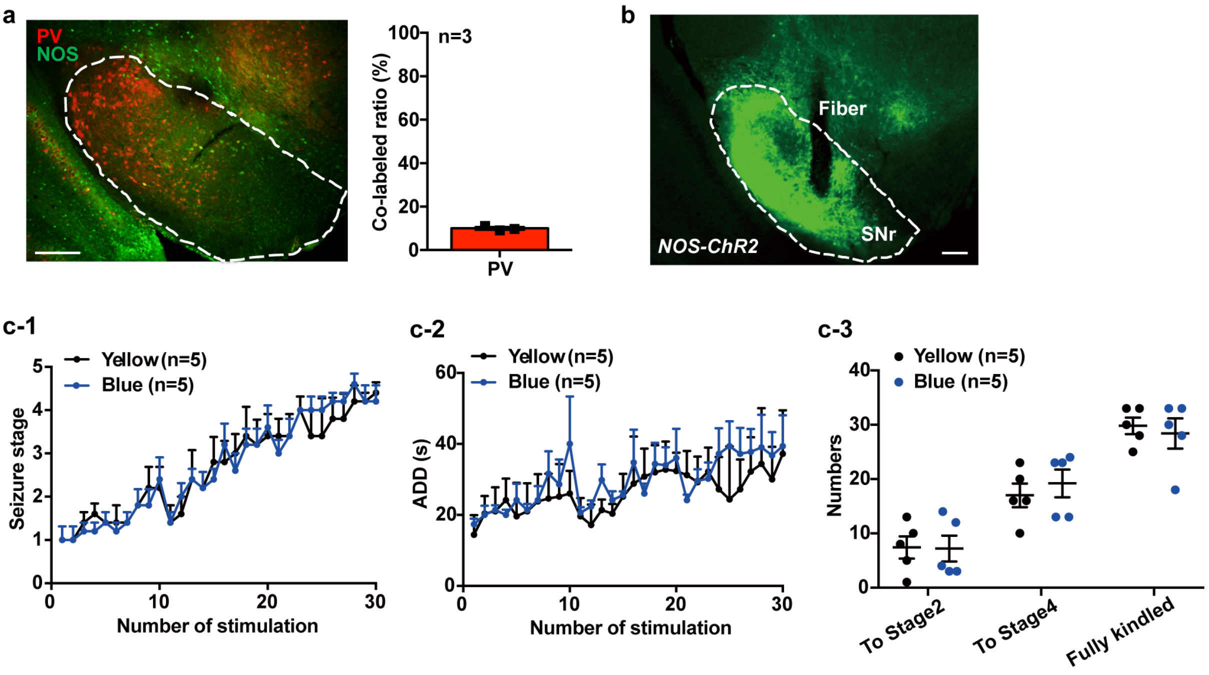

Supplementary Figure 3. Activation of SNr NOS neurons has no effects on kindling progression in hippocampal kindling model. (**a**) Representative image of the SNr showing PV and NOS neurons from one wildtype mouse; Scale bar, 100 µm. Right panel, statistical data of co-labeled ratio from 3 wildtype mice. (**b**) Representative image of the SNr from a *NOS-ChR2*^{SNr} mouse. Scale bar, 100 µm. (**c**) Effects of optogenetic activation of SNr NOS neurons on the development of seizure stage (**c-1**), afterdischarge duration (ADD, **c-2**), and number of stimulations needed to reach each seizure stage (**c-3**) in hippocampal kindling model. The number of mice used in each group is indicated in figure. All the data are presented as mean ± S.E.M.. Source data are provided as a Source Data file.

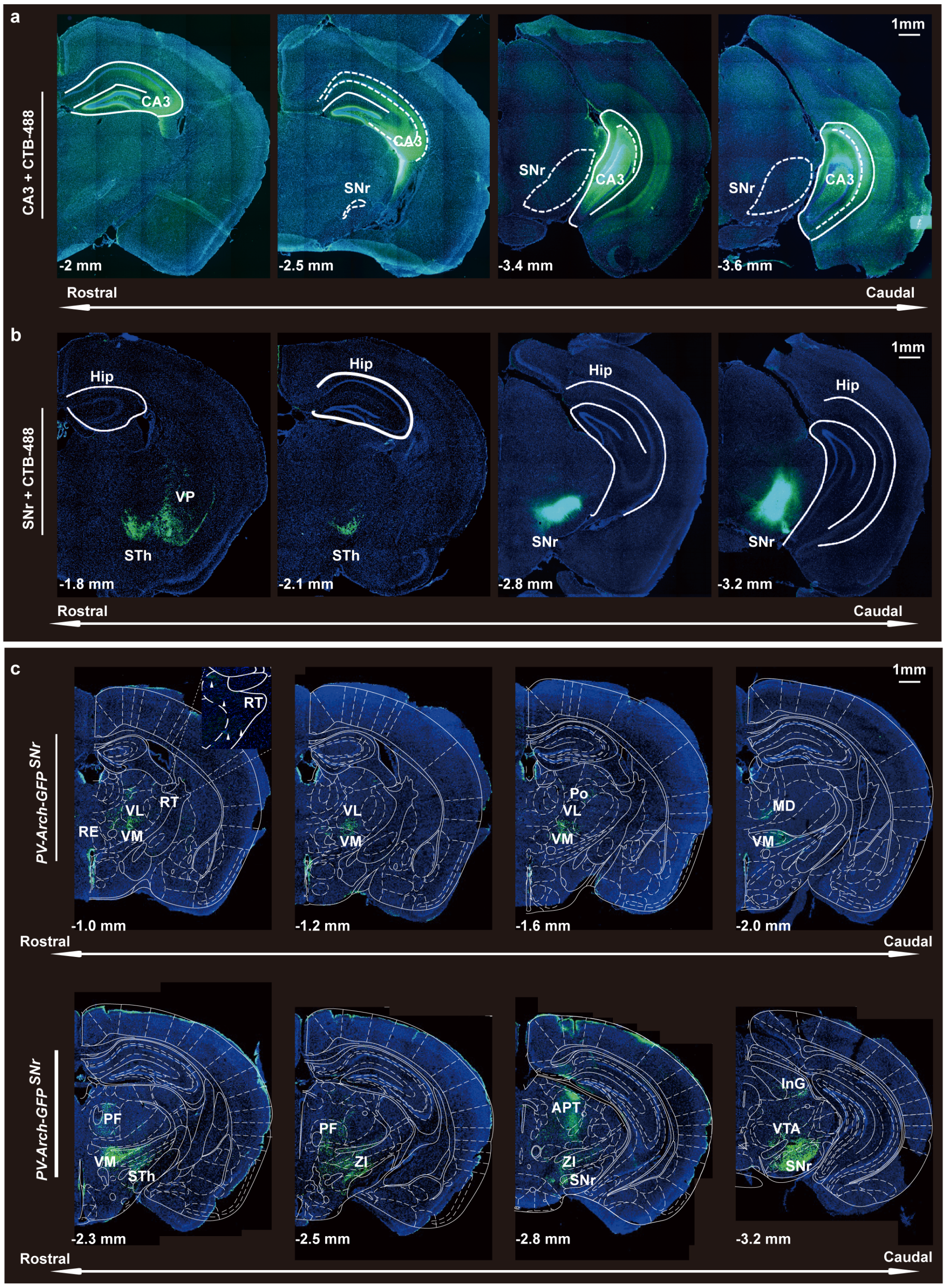

Supplementary Figure 4. There is no direct projection between the hippocampus and SNr. (**a**) Series of coronal sections showing CTB conjugated to AlexaFluor-488 (CTB-488, green) injection into ipsilateral CA3 from a representative wildtype mouse. (**b**) Series of coronal sections showing CTB-488 injection into ipsilateral SNr from a representative wildtype mouse. Hip, hippocampus. SNr, substantia nigra pars reticulata. STh, subthalamic nucleus. VP, ventral pallidum. **(c)** Primary outputs of SNr PV neurons. Series of coronal sections from a representative *PV-Arch*^{SNr} mouse showing the major outputs of the SNr. APT, anterior pretectal nucleus. InG, intermediate gray layer of the superior colliculus. MD, mediodorsal thalamic nucleus. PF, parafascicular nucleus. Po, posterior thalamic nuclear group. RE, reuniens thalamic nucleus. RT, reticular thalamic nucleus. SNr, substantia nigra pars reticulata. STh, subthalamic nucleus. VL, ventrolateral thalamic nucleus. VM, ventromedial thalamic nucleus. VTA, ventral tegmental aera. ZI, zona incerta.

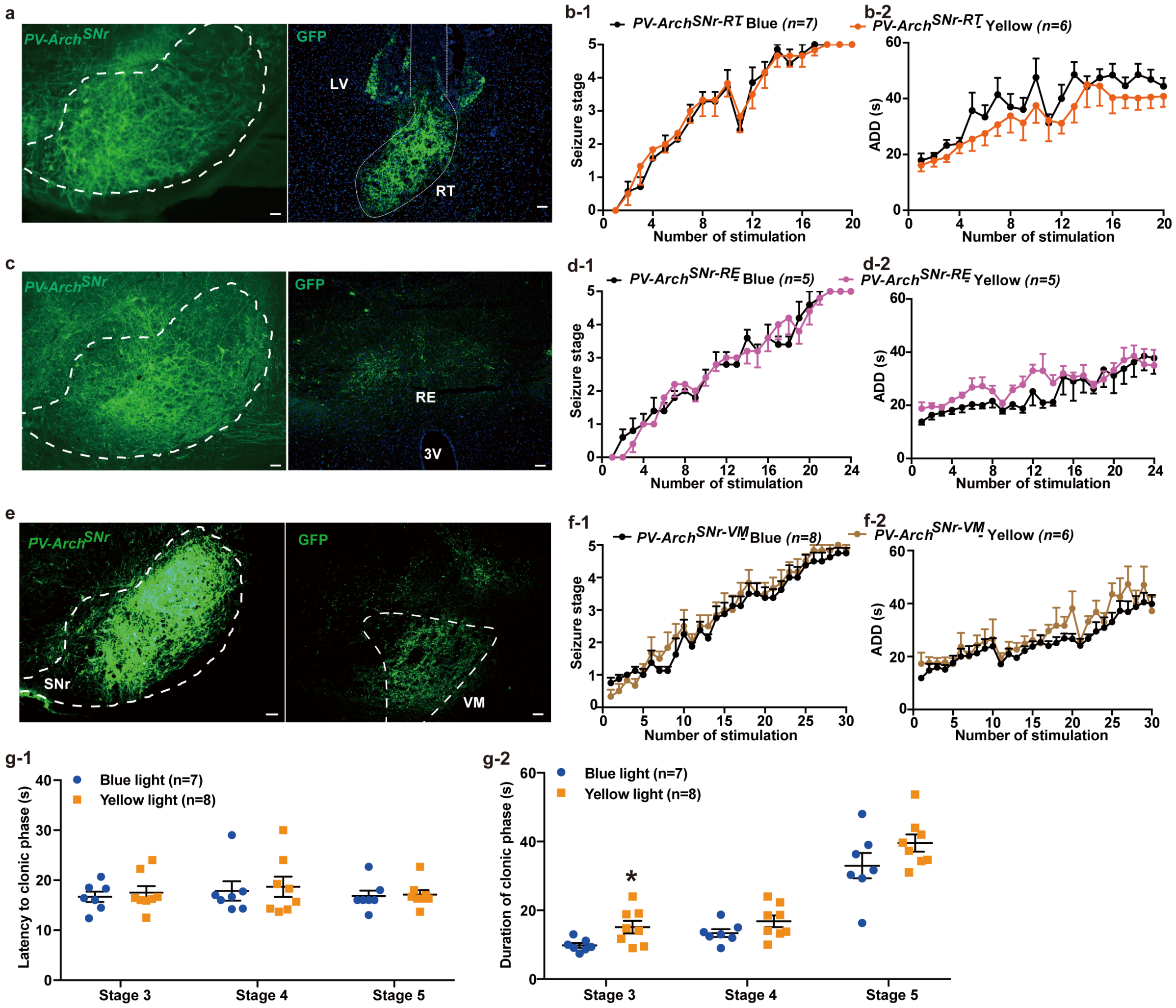

Supplementary Figure 5. Optogenetic inhibition of SNr-RT, SNr-RE or SNr-VM pathway has no effect on kindling progression. (**a, c, e**) Representative images showing the expression of Arch-GFP soma in the SNr, and Arch-GFP axon fibers within the RT **(a)**, RE **(c)** or VM **(e)** from *PV-Arch*^{SNr} mice. Scale bar, 50 µm. (**b, d, f**) Effects of optogenetic inhibition of SNr-RT (**b**), SNr-RE (**d**), SNr-VM **(f)** PV projections on the development of seizure stage and afterdischarge duration (ADD) in hippocampal kindling model. **(g)** Effects of optogenetic inhibition of SNr-VM PV projections on the latency to clonic phase **(g-1)** and average clonic duration **(g-2)** in seizure stage 3-5. Student's t test was used, **P*<0.05. The number of mice used in each group is indicated in figure. All the data are presented as mean ± S.E.M.. Source data are provided as a Source Data file.

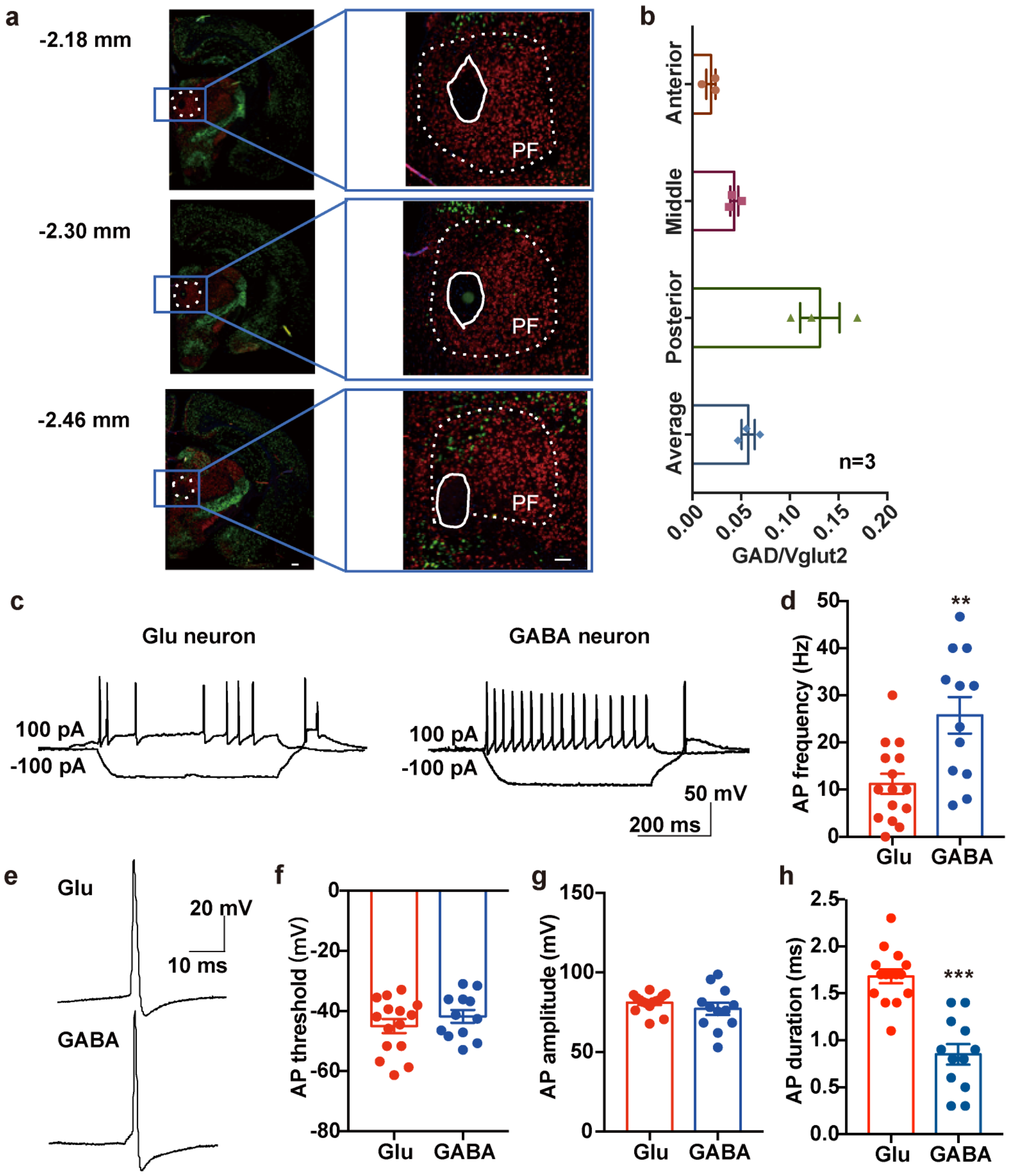

Supplementary Figure 6. Electrophysiological feature of glutamatergic and GABAergic neurons in posterior PF. (**a**) Series of coronal sections from a representative wildtype mouse showing GAD$^+$ (green) and Vglut2$^+$ (red) neurons in the PF by using fluorescence *in situ* hybridization (RNAscope Multiplex Fluorescent Reagent Kit, Probe-GAD1 and Probe-Slc17a6; Advanced Cell Diagnostics, Inc.). Scale bar, 100 μm. (**b**) Proportion quantification of the PF GABAergic neurons and glutamatergic neurons from 3 wildtype mice. **(c)** Representative traces of voltage responses of a PF glutamatergic or GABAergic neuron to hyperpolarizing and depolarizing current pulses. **(d)** Action potential (AP) frequency of PF glutamatergic and GABAergic neurons to 100-pA depolarizing current pulses. **(e-h)** Action potential (AP) firing properties, including representative AP traces **(e)**, AP initiation threshold **(f)**, AP amplitude **(g)**, and AP duration **(h)** of glutamatergic and GABAergic neurons in the PF (n=15 for Glu, n=12 for GABA, from 8 slices of 6 mice). $^{**}P<0.01$, $^{***}P<0.001$, Student's t test was used. All the data are presented as mean ± S.E.M.. Source data are provided as a Source Data file.

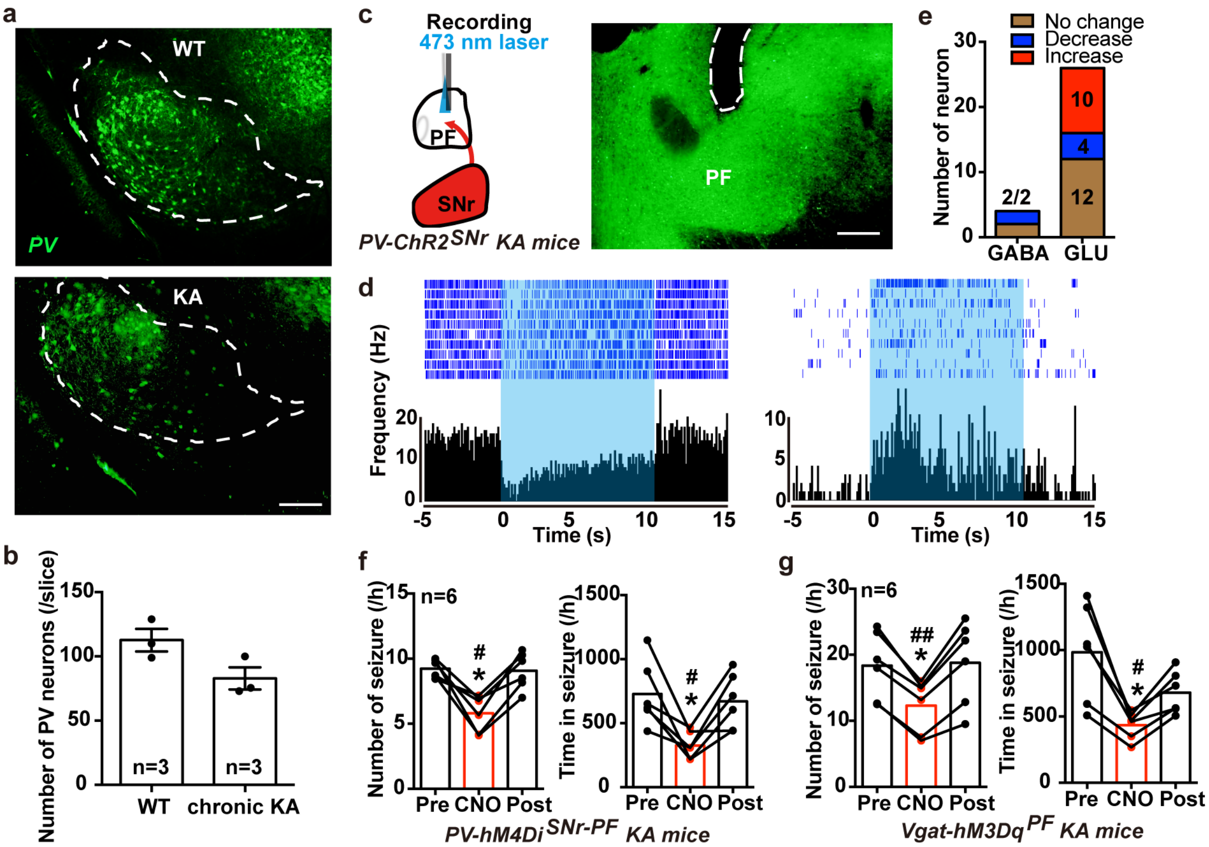

Supplementary Figure 7. SNr-PF circuit is involved in seizure control in KA-induced chronic epileptic model. (**a**, **b**) Representative immunohistochemistry images (**a**) and statistical data (**b**) showing the number of PV neurons in the SNr from wildtype and KA mice. Bar, 200 µm. (**c**) Scheme of experiment for *in vivo* single-unit recordings in the PF of awake $PV\text{-}ChR2^{SNr\text{-}PF}$ chronic epileptic mice. Bar, 200 µm. (**d**) Representative peri-event raster histograms showing the firing rate of PF GABAergic and glutamatergic neurons in response to photo-activation of SNr-PF PV projections; (**e**) Quantification of the number of PF GABAergic and glutamatergic neurons in response to photo-activation of SNr-PF PV projections from 3 $PV\text{-}ChR2^{SNr\text{-}PF}$ mice. (**f**) Effects of chemogenetic inhibition of SNr-PF PV projection in $PV\text{-}hM4Di^{SNr\text{-}PF}$ mice on the number and duration of seizures in KA-induced chronic epileptic model. Wilcoxon matched-pairs test, $^{*}P<0.05$ compared to Pre; $^{\#}P<0.05$ compared to Post. (**g**) Effects of chemogenetic activation of PF GABAergic neurons in $PV\text{-}hM3Dq^{PF}$ mice on the number and duration of seizures in KA-induced chronic epileptic model. Wilcoxon matched-pairs test, $^{*}P<0.05$ compared to Pre; $^{\#}P<0.05$ compared to Post. The number of neurons and mice used in each group is indicated in figure. All the data are presented as mean ± S.E.M.. Source data are provided as a Source Data file.

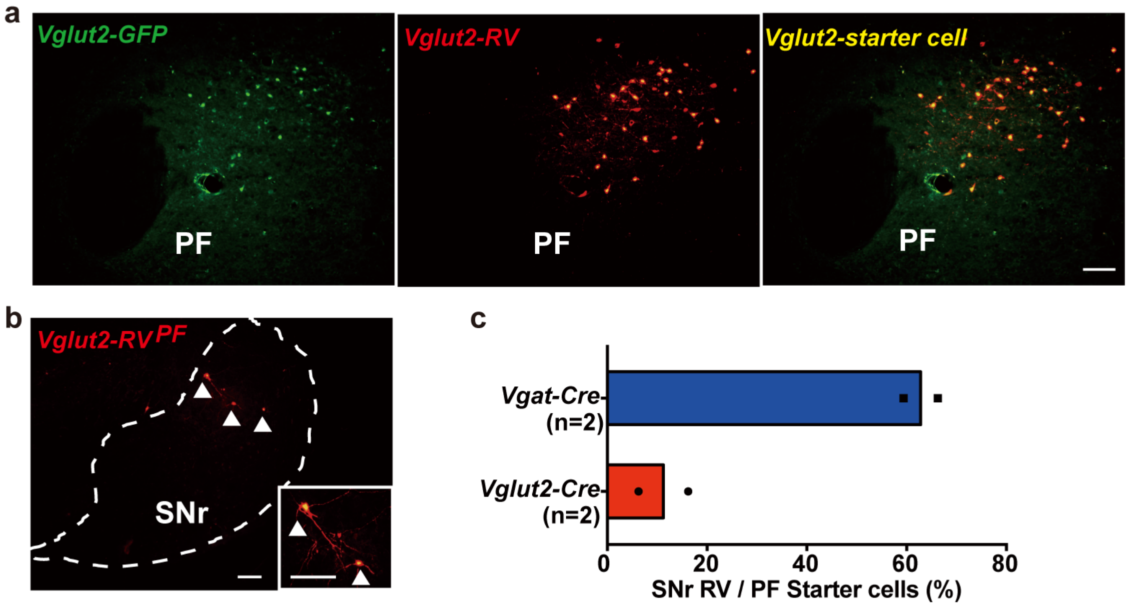

Supplementary Figure 8. **SNr PV neurons limitedly innervate PF glutamatergic neurons.** (**a**) PF retrograde monosynaptic tracing in *Vglut2-Cre* mice using a modified rabies virus system. Representative images of the PF injected with the helper virus and rabies virus: showing PF glutamatergic neurons infected by the helper virus (GFP), rabies virus (mCherry), or both viruses. The double-infected (yellow) glutamatergic neurons represent the starter cells. Scale bar, 100 μm. (**b**) Representative images of the SNr from the same mouse as that in **a**, showing the labeling of neurons by the monosynaptic retrograde spread of rabies virus expressing mCherry. Scale bar, 100 μm. (**c**) Quantification of the percentage of rabies virus in the SNr from PF GABAergic and glutamatergic neurons. Source data are provided as a Source Data file.

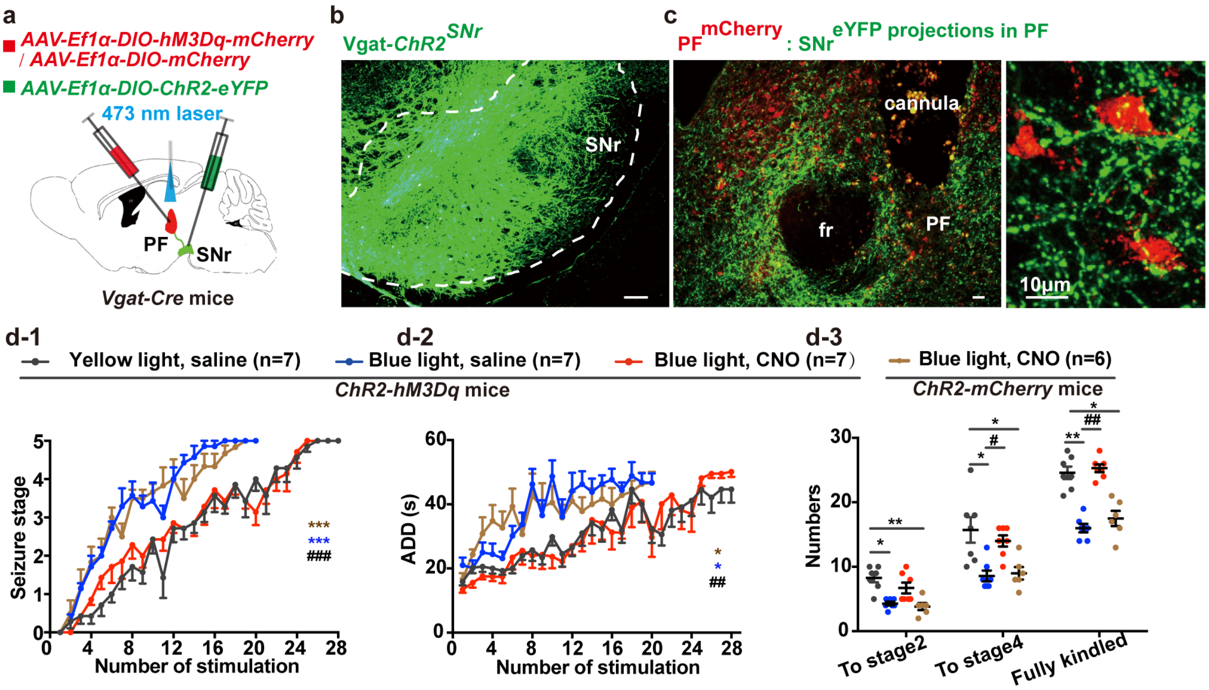

Supplementary Figure 9. Chemogenetic activation of PF GABAergic neurons blocks pro-epileptic effects of optogenetic activation of SNr-PF GABAergic projections in hippocampal kindling model. (**a**) Schematic of experiment for viral injection and photo-stimulation in *Vgat-Cre* mice. (**b**) Representative image showing the expression of ChR2-eYFP soma in the SNr. Scale bar, 100 μm. (**c**) Representative images from the same mouse in **b**, showing SNr-PF GABAergic neuronal projections labeled with ChR2-eYFP (green) and PF GABAergic neurons labeled with mCherry (red). Scale bar, 50 μm. (**d**) Effects of optogenetic activation of SNr-PF GABAergic projections on the development of seizure stage (**d-1**), afterdischarge duration (ADD, **d-2**) and number of stimulations needed to reach each seizure stage (**d-3**) in hippocampal kindling model, in the presence of chemogenetic activation of PF GABAergic neurons. Align-and-rank data for a nonparametric ANOVA followed by *post hoc* Dunn's test was used for **d-1,** General linear model with *post hoc* Dunn's test for multiple comparisons was used in **d-2**, and Kruskal-Wallis test with *post hoc* Dunn's test for multiple comparisons was used in **d-3**, **P*<0.05, ***P*<0.01, ****P*<0.001 compared with yellow light with saline, $^{\#}P$<0.05, $^{\#\#}P$<0.01, $^{\#\#\#}P$<0.001 compared with blue light with saline. The number of mice used in each group is indicated in figure. All the data are presented as mean ± S.E.M.. Source data are provided as a Source Data file.

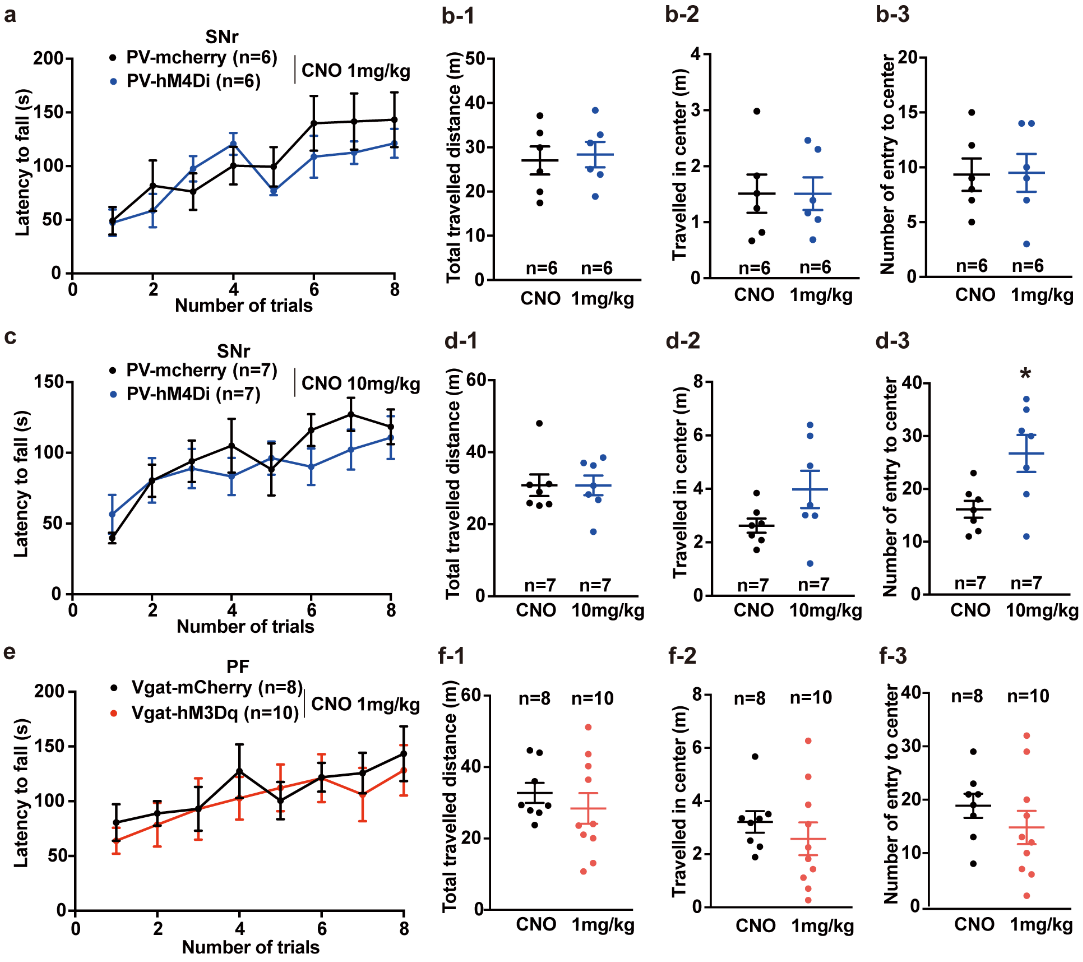

Supplementary Figure 10. Chemogenetic modulation of SNr PV neurons or PF GABAergic neurons at low dose does not inference the motor functions. (**a**) Chemogenetic inhibition of SNr PV neurons at dose of 1 mg/kg CNO does not inference motor coordination in rotarod test. (**b**) Chemogenetic inhibition of SNr PV neurons at dose of 1 mg/kg CNO does not inference motor behavior, including total travel distance (**b-1**), travel distance in center (**b-2**) and number of entry into center (**b-3**), in 5-min open-field test. (**c**) Chemogenetic inhibition of SNr PV neurons at dose of 10 mg/kg CNO does not inference motor coordination in rotarod test. (**d**) Effects of chemogenetic inhibition of SNr PV neurons at dose of 10 mg/kg CNO on motor behavior, including total travel distance (**d-1**), travel distance in center (**d-2**) and number of entry into center (**d-3**), in 5-min open-field test. (**e**) Chemogenetic activation of PF GABAergic neurons at dose of 1 mg/kg CNO does not inference motor learning in rotarod test. (**f**) Chemogenetic activation of PF GABAergic neurons at dose of 1 mg/kg CNO does not inference motor behavior, including total travel distance (**f-1**), travel distance in center (**f-2**) and number of entry into center (**f-3**), in 5-min open-field test. The number of mice used in each group is indicated in figure. All the data are presented as mean ± S.E.M.. Source data are provided as a Source Data file.

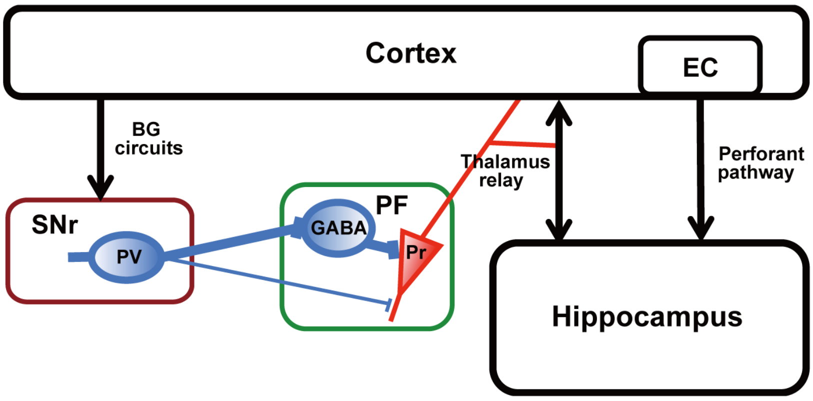

Supplementary Figure 11. Summary of a disinhibitory nigra-parafascicular neural circuit in seizure in temporal lobe epilepsy.

Supplementary Table 1. Detailed information for statistics

Figure	Statistic test	Statistic parameter
**Fig.1f**	Align-and-rank data for a nonparametric ANOVA	F=59.131, P<0.0001
**Fig.1g**	General linear model with repeated measures	F=9.765, P=0.007
**Fig.1h**	Mann-Whitney U test	U=3, P=0.0014 for To stage 2;<br>U=1.5, P=0.0005 for To stage 4;<br>U=0, P=0.0002 for Fully kindled;
**Fig.1j**	Unpaired t test	t=2.195, P=0.0455
**Fig.2d**	Align-and-rank data for a nonparametric ANOVA	F=29.969, P<0.0001
**Fig.2e**	General linear model with repeated measures	F=8.635, P=0.011
**Fig.2f**	Mann-Whitney U test	U=19, P=0.1740 for To stage 2;<br>U=3, P=0.0009 for To stage 4;<br>U=0, P=0.0002 for Fully kindled;
**Fig.2g**	Mann-Whitney U test	U=28, P=0.7030
**Fig.2i**	Unpaired t test	t=2.524, P=0.0243
**Fig.3b**	Kruskal-Wallis test with post hoc Dunn's tests for multiple comparisons	KW statistic=9.305, P=0.0053;<br>For Colunm A vs Colunm B, P=0.5926;<br>For Colunm A vs Colunm C, P=0.0217;<br>For Colunm B vs Colunm C, P=0.0044;
**Fig.3c**	Kruskal-Wallis test with post hoc Dunn's tests for multiple comparisons	KW statistic=12.15, p=0.0005;<br>For Colunm A vs Colunm B, P=0.9017;<br>For Colunm A vs Colunm C, P=0.0035;<br>For Colunm B vs Colunm C, P=0.0023;
**Fig.3d**	Kruskal-Wallis test with post hoc Dunn's tests for multiple comparisons	KW statistic=10.26, p=0.0026;<br>For Colunm A vs Colunm B, P=0.7256;<br>For Colunm A vs Colunm C, P=0.0036;<br>For Colunm B vs Colunm C, P=0.0109;
**Fig.3e**	Kruskal-Wallis test with post hoc Dunn's tests for multiple comparisons	KW statistic=7.537, p=0.0169;<br>For Colunm A vs Colunm B, P=0.6453;<br>For Colunm A vs Colunm C, P=0.0375;<br>For Colunm B vs Colunm C, P=0.0106;
**Fig.3i**	Wilcoxon matched-pairs signed rank test	P=0.0312 compared with Pre, P=0.0312 compared with Post for number of FS<br>P=0.0312 compared with Pre, P=0.0312 compared with Post for time in FS
**Fig.3j**	Wilcoxon matched-pairs signed rank test	P=0.0312 compared with Pre, P=0.0938 compared with Post for number of GS<br>P=0.0312 compared with Pre, P=0.0938 compared with Post for time in GS
**Fig.3k**	Wilcoxon matched-pairs signed rank test	P>0.9999 compared with Pre, P>0.9999 compared with Post for number of FS<br>P=0.1875 compared with Pre, P=0.8125 compared with Post for time in FS
**Fig.3l**	Wilcoxon matched-pairs signed rank test	P=0.7500 compared with Pre, P=0.3125 compared with Post for number of GS<br>P=0.1875 compared with Pre, P=0.4375 compared with Post for time in GS
**Fig.3n**	Wilcoxon matched-pairs signed rank test	P=0.0312 compared with Pre, P=0.1250 compared with Post for number of FS<br>P=0.0312 compared with Pre, P=0.1250 compared with Post for time in FS

**Fig.3o**	Wilcoxon matched-pairs signed rank test	P=0.0312 compared with Pre, P=0.5000 compared with Post for number of GS<br>P=0.0312 compared with Pre, P=0.1250 compared with Post for time in GS
**Fig. 4c**	Align-and-rank data for a nonparametric ANOVA	F=35.589, P<0.0001
**Fig. 4d**	General linear model with repeated measures	F=17.488, P=0.001
**Fig. 4e**	Mann-Whitney U test	U=10, P=0.0228 for To stage 2;<br>U=6, P=0.0036 for To stage 4;<br>U=0, P=0.0002 for Fully kindled;
**Fig. 4g**	Align-and-rank data for a nonparametric ANOVA followed by post hoc Dunn's test for multiple comparisons	F=12.053, P<0.0001<br>For Yellow vs Blue, P=0.002;<br>For Yellow vs Blue+Bic, P=0.901;<br>For Blue vs Blue+Bic, P=0.001;
**Fig. 4h**	General linear model with repeated measures followed by post hoc Dunn's test for multiple comparisons	F=6.293 P=0.008;<br>For Yellow vs Blue, P=0.026;<br>For Yellow vs Blue+Bic, P=0.929<br>For Blue vs Blue+Bic, P=0.012
**Fig. 4i**	Kruskal-Wallis test with post hoc Dunn's tests for multiple comparisons	For To stage 2, KW statistic=12.39, P=0.0004;<br>For Column A vs Column B, P=0.0050;<br>For Column A vs Column C, P>0.9999;<br>For Column B vs Column C, P=0.0097;<br><br>For To stage 4, KW statistic=6.853, P=0.0262;<br>For Column A vs Column B, P=0.0279;<br>For Column A vs Column C, P=0.8948;<br>For Column B vs Column C, P=0.3561;<br><br>For Fully kindled, KW statistic=8.22, p=0.0111;<br>For Column A vs Column B, P=0.0347;<br>For Column A vs Column C, P>0.9999;<br>For Column B vs Column C, P=0.0443;
**Fig. 5b**	Wilcoxon matched-pairs signed rank test	P=0.0312 compared with Pre, P=0.0938 compared with Post
**Fig. 6d**	Align-and-rank data for a nonparametric ANOVA	F=20.130, P<0.0001
**Fig. 6e**	General linear model with repeated measures	F=9.551, P=0.008
**Fig. 6f**	Mann-Whitney U test	U=18, P=0.0853 for To stage 2;<br>U=2.5, P=0.0011 for To stage 4;<br>U=5, P=0.0031 for Fully kindled;
**Fig. 6j**	Align-and-rank data for a nonparametric ANOVA followed by post hoc Dunn's test for multiple comparisons	F=9.800, P=0.001;<br>For Yellow vs Blue, P=0.004;<br>For Yellow vs Blue+Bicuculline, P=0.9996;<br>For Blue vs Blue+ Bicuculline, P=0.003;
**Fig. 6k**	General linear model with repeated measures followed by post hoc Dunn's test for multiple comparisons	F=4.363 P=0.029;<br>For Yellow vs Blue, P=0.040;<br>For Yellow vs Blue+ Bicuculline, P=0.566<br>For Blue vs Blue+ Bicuculline, P=0.012
**Fig. 6l**	Kruskal-Wallis test with post hoc Dunn's tests for multiple comparisons	For To stage 2, KW statistic=6.397, P=0.0351;<br>For Column A vs Column B, P=0.0463;<br>For Column A vs Column C, P=0.7261;<br>For Column B vs Column C, P=0.0191;<br><br>For To stage 4, KW statistic=11.7, P=0.0007;<br>For Column A vs Column B, P=0.0121;

		For Colunm A vs Colunm C, P=0.4489;<br>For Colunm B vs Colunm C, P=0.0011;<br><br>For Fully kindled, KW statistic=11.89, P=0.0006;<br>For Colunm A vs Colunm B, P=0.0025;<br>For Colunm A vs Colunm C, P=0.9310;<br>For Colunm B vs Colunm C, P=0.0033;
**Fig. 6n**	Mann-Whitney U test	U=21, P=0.0364
**Fig. 6o**	Mann-Whitney U test	U=30, P=0.1734
**Fig. 6p**	Mann-Whitney U test	U=22, P=0.0422
**Fig. 6q**	Chi-aquare test	Chi-aquare=2.967, P=0.0850
**Fig. 7f**	Align-and-rank data for a nonparametric ANOVA	F=22.383, P<0.0001
**Fig. 7g**	General linear model with repeated measures	F=9.579, P=0.007
**Fig. 7h**	Mann-Whitney U test	U=4.5, P=0.0003 for To stage 2;<br>U=12, P=0.0048 for To stage 4;<br>U=13, P=0.0071 for Fully kindled;
**Fig. 7j**	Align-and-rank data for a nonparametric ANOVA	F=41.696, P<0.0001
**Fig. 7k**	General linear model with repeated measures	F=12.275, P=0.003
**Fig. 7l**	Mann-Whitney U test	U=5, P=0.0010 for To stage 2;<br>U=13, P=0.0139 for To stage 4;<br>U=6, P=0.0012 for Fully kindled;
**Fig. S1h-1**	Align-and-rank data for a nonparametric ANOVA followed by post hoc Dunn's test for multiple comparisons	F=12.004, P<0.0001<br>For Yellow vs Blue IMM, P=0.014;<br>For Yellow vs Blue Pre, P=0.125;<br>For Yellow vs Blue Delay, P=0.278;
**Fig. S1h-2**	General linear model with repeated measures post hoc Dunnett's test for multiple comparisons	F=4.865, P=0.008<br>For Yellow vs Blue IMM, P=0.013;<br>For Yellow vs Blue Pre, P=0.999;<br>For Yellow vs Blue Delay, P=987;
**Fig. S1h-3**	Kruskal-Wallis test with post hoc Dunn's tests for multiple comparisons	For To stage 2, KW statistic=7.114, P=0.0684;<br>For Colunm A vs Colunm B, P=0.1409;<br>For Colunm A vs Colunm C, P=0.4756;<br>For Colunm A vs Colunm D, P=0.2805;<br><br>For To stage 4, KW statistic=19.42, P=0.0002;<br>For Colunm A vs Colunm B, P=0.0098;<br>For Colunm A vs Colunm C, P=0.3700;<br>For Colunm A vs Colunm D, P=0.0842;<br><br>For Fully kindled, KW statistic=14.94, P=0.0019;<br>For Colunm A vs Colunm B, P=0.0120;<br>For Colunm A vs Colunm C, P=0.4784;<br>For Colunm A vs Colunm D, P=0.2463;
**Fig. S1i**	Mann-Whitney U test	U=25.5, P=0.5078
**Fig. S3c-1**	Align-and-rank data for a nonparametric ANOVA	F=0.139, P=0.719
**Fig. S3c-2**	General linear model with repeated measures	F=0.767, P=0.407
**Fig. S3c-3**	Mann-Whitney U test	U=12, P=0.9524 for To stage 2;<br>U=9, P=0.5794 for To stage 4;<br>U=12, P>0.9999 for Fully kindled;

**Fig. S5b-1**	Align-and-rank data for a nonparametric ANOVA	F=0.397, P=0.542
**Fig. S5b-2**	General linear model with repeated measures	F=1.488, P=0.248
**Fig. S5d-1**	Align-and-rank data for a nonparametric ANOVA	F=0.499, P=0.500
**Fig. S5d-2**	General linear model with repeated measures	F=1.6510, P=0.235
**Fig. S5f-1**	Align-and-rank data for a nonparametric ANOVA	F=0.252, P=0.625
**Fig. S5f-2**	General linear model with repeated measures	F=1.805, P=0.204
**Fig. S5g-1**	Mann-Whitney U test	For stage 3, U=15.5, P=0.8023<br>For stage 4, U=27.5, P=0.9800<br>For stage 5, U=18, P=0.2586
**Fig. S5g-2**	Mann-Whitney U test	For stage 3, U=9, P=0.0289<br>For stage 4, U=15, P=0.1520<br>For stage 5, U=15, P=0.1520
**Fig. S6d**	Student's t test	T=3.462, df=25, P=0.0019
**Fig. S6f**	Student's t test	T=0.9829, df=25, P=0.3351
**Fig. S6g**	Student's t test	T=1.02, df=25, P=0.3174
**Fig. S6h**	Student's t test	T=6.504, df=25, P<0.0001
**Fig.S7f**	Wilcoxon matched-pairs signed rank test	P=0.0312 compared with Pre, P=0.0312 compared with Post for number of seizures<br>P=0.0312 compared with Pre, P=0.0312 compared with Post for time in seizure
**Fig.S7g**	Wilcoxon matched-pairs signed rank test	P=0.0312 compared with Pre, P=0.0014 compared with Post for number of seizures<br>P=0.0312 compared with Pre, P=0.0312 compared with Post for time in seizure
**Fig.S9d-1**	Align-and-rank data for a nonparametric ANOVA followed by post hoc Dunn's test for multiple comparisons	F=34.827, P<0.0001<br>For Group 1 vs Group 2, P<0.0001;<br>For Group 1 vs Group 3, P=0.512;<br>For Group 1 vs Group 4, P<0.0001;<br>For Group 2 vs Group 3, P<0.0001;
**Fig.S9d-2**	general linear model with repeated measures followed by post hoc Dunn's test for multiple comparisons,	F=7.501, P=0.001<br>For Group 1 vs Group 2, P=0.010;<br>For Group 1 vs Group 3, P=0.913;<br>For Group 1 vs Group 4, P=0.022;<br>For Group 2 vs Group 3, P=0.005;
**Fig.S9d-3**	Kruskal-Wallis test with post hoc Dunn's tests for multiple comparisons,	For To stage 2, KW statistic=16.23, P=0.0010<br>For Group 1 vs Group 2, P=0.0142;<br>For Group 1 vs Group 3, P>0.9999;<br>For Group 1 vs Group 4, P=0.0040;<br>For Group 2 vs Group 3, P=0.2163;<br><br>For To stage 4, KW statistic=15.04, P=0.0018;<br>For Group 1 vs Group 2, P=0.0131;<br>For Group 1 vs Group 3, P>0.9999;<br>For Group 1 vs Group 4, P=0.0412;<br>For Group 2 vs Group 3, P=0.0312;<br><br>For Fully kindled, KW statistic=19.84, P=0.0002;<br>For Group 1 vs Group 2, P=0.0056;<br>For Group 1 vs Group 3, P>0.9999;<br>For Group 1 vs Group 4, P=0.0460;<br>For Group 2 vs Group 3, P=0.0019;

**Fig. S10a**	General linear model with repeated measures	F=0.312, P=0.589
**Fig. S10b-1**	Mann-Whitney U test	U=16, P=0.8182
**Fig. S10b-2**	Mann-Whitney U test	U=18, P>0.9999
**Fig. S10b-3**	Mann-Whitney U test	U=17, P=0.8983
**Fig. S10c**	General linear model with repeated measures	F=0.275, P=0.610
**Fig. S10d-1**	Mann-Whitney U test	U=21, P=0.7104
**Fig. S10d-2**	Mann-Whitney U test	U=12, P=0.1282
**Fig. S10d-3**	Mann-Whitney U test	U=8, P=0.0350
**Fig. S10e**	General linear model with repeated measures	F=0.158, P=0.696
**Fig. S10f-1**	Mann-Whitney U test	U=27, P=0.2743
**Fig. S10f-2**	Mann-Whitney U test	U=26, P=0.2370
**Fig. S10f-3**	Mann-Whitney U test	U=25.5, P=0.2102
