# Supplementary material for: A disinhibitory nigra-parafascicular pathway amplifies seizure in temporal lobe epilepsy
Source: Nat Commun. 2020 Feb 17;11:923. doi: 10.1038/s41467-020-14648-8 (PMC7026152; doi:10.1038/s41467-020-14648-8)
Supplement: Supplementary file 3 — Reporting Summary [file 41467_2020_14648_MOESM3_ESM.pdf]

nature research

Corresponding author(s): Zhong Chen
Last updated by author(s): Jan 21, 2020

Reporting Summary

Nature Research wishes to improve the reproducibility of the work that we publish. This form provides structure for consistency and transparency in reporting. For further information on Nature Research policies, see Authors & Referees and the Editorial Policy Checklist .

Statistics

For all statistical analyses, confirm that the following items are present in the figure legend, table legend, main text, or Methods section.

n/a	Confirmed	
☐	☒	The exact sample size (n) for each experimental group/condition, given as a discrete number and unit of measurement
☐	☒	A statement on whether measurements were taken from distinct samples or whether the same sample was measured repeatedly
☐	☒	The statistical test(s) used AND whether they are one- or two-sided<br>*Only common tests should be described solely by name; describe more complex techniques in the Methods section.*
☐	☒	A description of all covariates tested
☐	☒	A description of any assumptions or corrections, such as tests of normality and adjustment for multiple comparisons
☐	☒	A full description of the statistical parameters including central tendency (e.g. means) or other basic estimates (e.g. regression coefficient) AND variation (e.g. standard deviation) or associated estimates of uncertainty (e.g. confidence intervals)
☐	☒	For null hypothesis testing, the test statistic (e.g. F, t, r) with confidence intervals, effect sizes, degrees of freedom and P value noted<br>*Give P values as exact values whenever suitable.*
☒	☐	For Bayesian analysis, information on the choice of priors and Markov chain Monte Carlo settings
☒	☐	For hierarchical and complex designs, identification of the appropriate level for tests and full reporting of outcomes
☒	☐	Estimates of effect sizes (e.g. Cohen's d, Pearson's r), indicating how they were calculated

Our web collection on statistics for biologists contains articles on many of the points above.

Software and code

Policy information about availability of computer code

Data collection	EEG was recorded with a digital amplifier (NuAmps, Neuroscan System, USA) or a PowerLab system (AD Instruments, Australia); The EEG recorded in the ventral hippocampus was analyzed off-line by a software package (Scan 4.5) in the Neuronscan System. Single-unit data recording was collected with the Cerebus acquisition system (Version 6.04, Blackrock Microsystems, USA); Recorded neuronal data were offline resorted by offline sorting software (version 3.3, Plexon Inc, USA) to confirm the quality of the recorded cells. Ca2+ signaling were recorded by the fiber photometry system (Nanjing Thinkertech, China); Peri-event histograms and autocorrelation analysis were performed to analyze sorted neuronal data by neuroexplorer 4.0 software (Nex Technologies Int Inc, USA); Locomotor behavioral analysis was conducted using ANY-maze (ANY maze Video Tracking System version 4.98, Stoelting Co., USA); In vitro electrophysiology was performed by using an EPC10 patch-clamp amplifier (HEKA Instruments; Germany); Confocal images were captured with Olympus FV-1000.
Data analysis	Statistical comparisons were performed using SPSS (version 17.0) or Prism (version 7.0) with appropriate inferential methods as indicated in the figure legends.

For manuscripts utilizing custom algorithms or software that are central to the research but not yet described in published literature, software must be made available to editors/reviewers. We strongly encourage code deposition in a community repository (e.g. GitHub). See the Nature Research guidelines for submitting code & software for further information.

Data

Policy information about availability of data

All manuscripts must include a data availability statement. This statement should provide the following information, where applicable:

- Accession codes, unique identifiers, or web links for publicly available datasets
- A list of figures that have associated raw data
- A description of any restrictions on data availability

The datasets generated during and/or analysed during the current study are available from the corresponding author upon reasonable request. The source data are provided as a Source Data file with the paper.

Field-specific reporting

Please select the one below that is the best fit for your research. If you are not sure, read the appropriate sections before making your selection.

- [x] Life sciences
- [ ] Behavioural & social sciences
- [ ] Ecological, evolutionary & environmental sciences

For a reference copy of the document with all sections, see nature.com/documents/nr-reporting-summary-flat.pdf

Life sciences study design

All studies must disclose on these points even when the disclosure is negative.

Sample size	No statistical methods were used to pre-determine sample size. The sample size was determined according to previous studies (PMID: 27929004, PMID: 28858623, PMID: 27908611)
Data exclusions	Only the mice with correct place of the electrodes implantation and the viral expression were taken into analysis. See page 28
Replication	We did the same experiments several times as indicated in the manuscript and got similar results.
Randomization	We allocate the mice into different group with their strains and their epileptogenetic sensitivity (afterdischarged threshold was used for grouping in the kindling model). The approach is to first adjust for the covariates and then normalize the residuals.
Blinding	The data collection and analysis were preformed by investigators blinded to the group allocation.

Reporting for specific materials, systems and methods

We require information from authors about some types of materials, experimental systems and methods used in many studies. Here, indicate whether each material, system or method listed is relevant to your study. If you are not sure if a list item applies to your research, read the appropriate section before selecting a response.

Materials & experimental systems

n/a	Involved in the study	
[ ]	[x]	Antibodies
[x]	[ ]	Eukaryotic cell lines
[x]	[ ]	Palaeontology
[ ]	[x]	Animals and other organisms
[x]	[ ]	Human research participants
[x]	[ ]	Clinical data

Methods

n/a	Involved in the study	
[x]	[ ]	ChIP-seq
[x]	[ ]	Flow cytometry
[x]	[ ]	MRI-based neuroimaging

Antibodies

Antibodies used	Primary antibodies used: Rabbit anti-GAD65/67 (Millipore AB1511), Rabbit anti-nNOS (ThermoFisher 61-7000), and Rabbit anti-PV (Swant PV27). Alexa-594 or Alexa-488 conjugated secondary fluorescent antibody (Jackson ImmunoResearch, donkey, 711-585-152 or 711-545-152). The dilutions of the antibodies in the manuscript is PBS.
Validation	The antibodies have been validated extensively. For anti-GAD65/67, please see references (PMID: 25294882, PMID: 28858623); For anti-PV, please see references (PMID: 29425490, PMID: 28858623). For anti-nNOS, please see reference (PMID:29686073, PMID:29576387)

Animals and other organisms

Policy information about studies involving animals; ARRIVE guidelines recommended for reporting animal research

Laboratory animals	Vgat-Cre mice (stock number: 016962), Vgat-ChR2-eYFP mice (stock number: 014548), PV-Cre mice (stock number: 008069), NOS-Cre mice (stock number: 017526) and Vglut2-Cre mice (stock number: 016963) were genotyped according to the protocols provided by Jackson Laboratories. All mice were bred onto a C57BL/6J genetic background. Male mice 2-4 months of age were used in all experiments. All mice were group-housed (4-6 per cage) prior to surgery under a 12 h light-dark cycle (light on from 8:00 a.m. to 8:00 p.m.) with ad libitum access to food and water. The mice were individually housed after surgery. All behavior experiments were performed each day between 10:00 a.m. and 7:00 p.m.
Wild animals	The study did not involved wild animals.
Field-collected samples	The study did not involved field-collected samples.
Ethics oversight	The use and care of the mice were in accordance with the guidelines of the Animal Advisory Committee of Zhejiang University

Note that full information on the approval of the study protocol must also be provided in the manuscript.
